# Resolution of lamellar macular hole after suprachoroidal triamcinolone injections for cystoid macular edema caused by retinal vein occlusion: A case report

**DOI:** 10.1097/MD.0000000000044647

**Published:** 2025-09-19

**Authors:** Sedra Abu Ghedda, Reem Farwati, Marwa Baba, Ameen Marashi

**Affiliations:** a Faculty of Medicine, University of Aleppo, Aleppo, Syria; b Marashi Eye Clinic, Aleppo, Syria.

**Keywords:** custom-made needle, lamellar hole, retinal vein occlusion, suprachoroidal, triamcinolone acetonide

## Abstract

**Rationale:**

Lamellar macular holes (LMHs) typically require surgical intervention, posing inherent risks. This case explores a non-surgical alternative: suprachoroidal triamcinolone injection, previously used for cystoid macular edema (CME) due to retinal vein occlusion, which unexpectedly resulted in LMH resolution. The significance lies in potentially expanding treatment options for LMH beyond surgery.

**Patient concerns:**

A 67-year-old female presented with bilateral visual impairment following retinal vein and artery occlusions. This was further complicated by a degenerative lamellar hole in the right eye identified via optical coherence tomography imaging, contributing to significantly reduced visual acuity in both eyes.

**Diagnoses:**

The patient’s history of retinal vascular occlusions led to the development of CME. optical coherence tomography revealed a degenerative lamellar hole in the right macula.

**Interventions:**

The patient received 2 suprachoroidal triamcinolone acetonide injections via a custom-made needle, initially intended to address the CME.

**Outcomes:**

Following the injections, the lamellar hole in the right eye resolved, and the patient experienced improvements, though the degree of visual acuity improvement is not fully specified in the abstract.

**Lesson:**

Suprachoroidal triamcinolone acetonide injections may offer a non-surgical treatment option for LMHs associated with CME secondary to retinal vein occlusion. Further research is needed to confirm these findings and determine the efficacy and safety of this approach in a larger patient population.

## 1. Introduction

Retinal vein occlusion (RVO) is a common retinal vascular disorder and the second most prevalent condition after diabetic retinopathy, often resulting in significant ocular complications.^[1]^ Among these complications, cystoid macular edema (CME) is a frequent and serious threat to vision in individuals with RVO.^[1,2]^

A lamellar macular hole (LMH) is characterized by tissue loss in the inner retinal layers, primarily affecting individuals aged 50 to 70. Its prevalence in the general population is between 1.1% and 3.6%.^[3]^ Most LMH cases have an unknown cause; however, there is a connection between LMH and conditions leading to chronic CME, such as RVO. To date, only 6 cases of LMH associated with RVO have been reported. Nevertheless, the etiology of this condition remains unclear.^[3,4]^ LMH can be identified through a dilated fundus examination.^[5]^ Fundus autofluorescence may assist in diagnosing LMH. A definitive diagnosis, however, relies on optical coherence tomography (OCT).^[3,4]^ Most LMH cases are initially asymptomatic and may not require treatment.^[2–4]^ However, symptoms can gradually develop and worsen over time, leading to reduced visual acuity, metamorphopsia, and scotomas.^[3,4]^ In symptomatic individuals, treatment is necessary; however, only surgical procedures are currently available for achieving successful remission. These procedures carry risks of complications such as cataract formation, non-closure of the hole, and development of full-thickness macular holes (FTMH).^[3,4]^ The suprachoroidal space has a unique anatomical position, closely connected to both the choroid and sclera. This characteristic may facilitate a slower washout rate and enhance medication absorption into target tissues. Pharmacokinetic studies in animals have shown that medication concentrations in the retina and choroid can be 12 times higher. Up to 3% of the drug enters the anterior chamber, reducing the risk of increased intraocular pressure (IOP), cataract formation, or exacerbation of existing glaucoma.^[6]^ The systemic withdrawal of corticosteroids from the suprachoroidal pathway is lower than that from other ocular injection routes. Human studies have assessed the safety and efficacy of suprachoroidal drug injections for diabetic macular edema (DME), RVO-related macular edema, and noninfectious uveitis.^[7,8]^ A phase 3 trial of preservative-free suprachoroidal delivery of triamcinolone acetonide (Clearside Biomedical, Alpharetta, GA, USA) led to FDA approval of a modified system (Xipere; Clearside Biomedical) due to its efficacy and excellent safety profile in treating noninfectious uveitis, with an IOP increase rate of 11.5%.^[9]^ Thus, the suprachoroidal route has the potential to provide therapeutic benefits with greater safety than posterior subtenon or intravitreal routes.^[10]^

In this study, we report the first successful case of treating LMH associated with RVO using a suprachoroidal injection of triamcinolone acetonide delivered via a custom-made needle.

In this study, we report the first successful case of treating LMH associated with RVO using a suprachoroidal injection of triamcinolone acetonide delivered with a custom-made needle.

## 2. Case presentation

**Figure 1.**
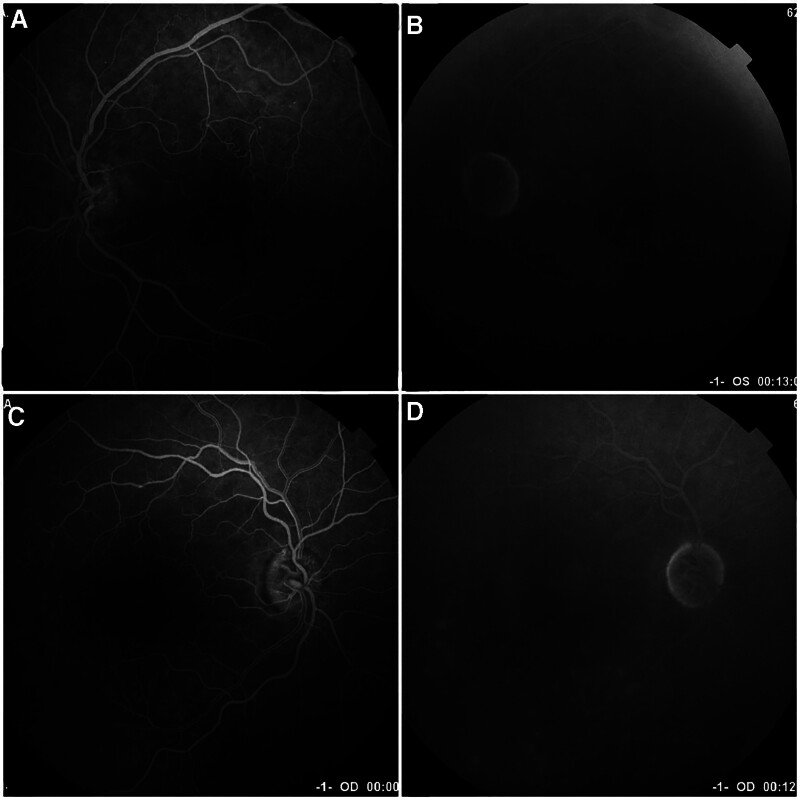
(A) Early phase FFA of the left eye shows normal retinal vascular circulation established after longstanding central artery occlusion. (B) Late phase FFA shows normal retinal vascular circulation established after longstanding central artery occlusion. (C) The early phase FFA photo of the right eye shows normal venous filling of the superior branch retinal vein, while the inferior retinal vein exhibits delayed venous filling with areas of capillary non-perfusion. Old laser retinal scars are visible as hyperfluorescent spots with hypofluorescent cores. (D) The late phase shows delayed filling and tortuosity of the inferior branch retinal vein, along with significant staining of the old laser scars with hypofluorescent cores. FFA = fundus fluorescein angiography.

**Figure 2.**
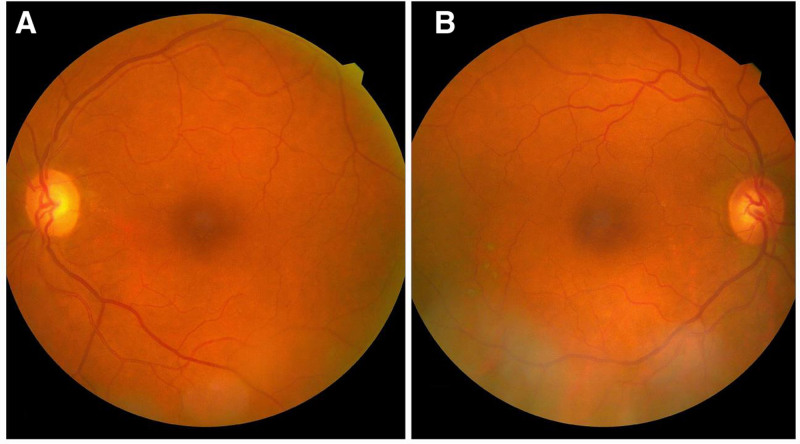
(A) The fundus image of the right eye shows old laser burns and inferior retinal dilation with a blunt foveal reflex. (B) The fundus image of the left eye shows thinning of the retinal artery and the tissue surrounding the macula.

**Figure 3.**
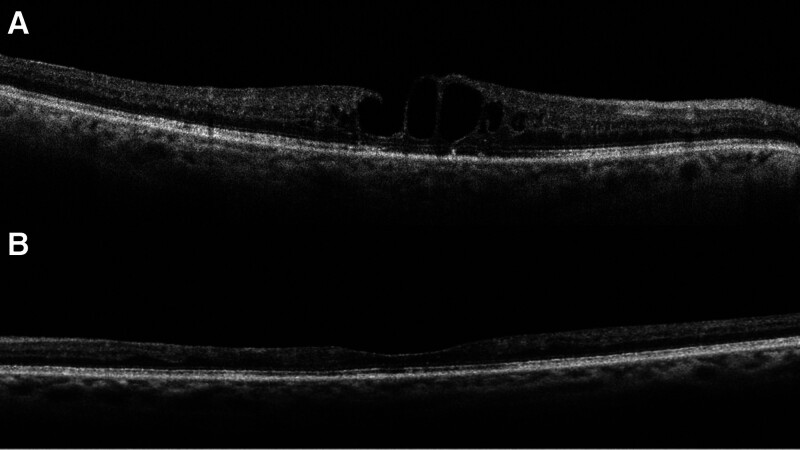
(A) The OCT of the right eye before the triamcinolone suprachoroidal injection shows a lamellar hole with intraretinal cystic changes, increased retinal thickness, and intact outer retinal layers due to branch retinal vein occlusion. (B) The OCT image of the left eye shows reduced retinal thickness due to an old ischemic central retinal artery occlusion. OCT = optical coherence tomography.

A 67-year-old phakic female with a history of hypertension presented to the clinic with bilateral visual impairment. She reported a decline in her vision, noting a previous episode of RVO in the right eye, which had been treated with laser therapy a year ago, as well as a retinal artery occlusion in the left eye (Fig. 1). Upon examination, the patient demonstrated a best visual acuity of counting fingers in the right eye and hand motion in the left eye, with intraocular pressure measuring 17 mm Hg bilaterally. Evaluation of the anterior segment revealed no significant findings. Fundus examination of the right eye showed evidence of old laser burns, inferior retinal vein dilation, and a blunt foveal reflex. In contrast, the left eye exhibited thinning of the retinal artery and surrounding macular tissue (Fig. 2). OCT of the right eye revealed a degenerative lamellar hole with cystic changes consistent with CME, while OCT of the left eye demonstrated reduced retinal thickness (Fig. 3).

## 3. Treatment

**Figure 4.**
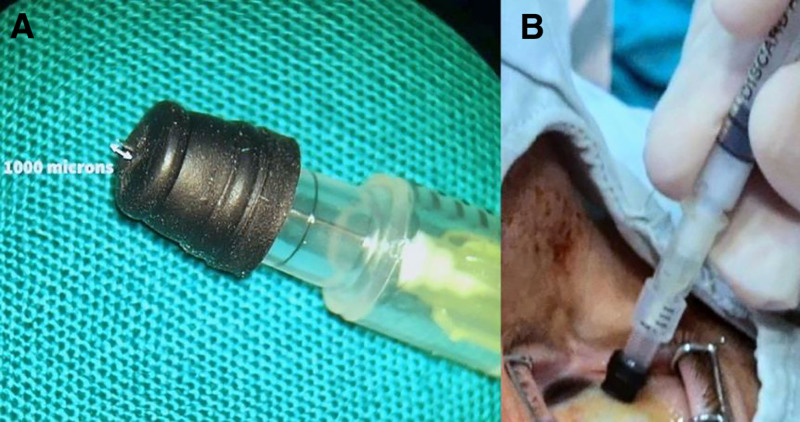
(A) The custom-designed needle developed and utilized by the author. (B) The technique employed for suprachoroidal injection using the custom-designed needle.

The author performed a suprachoroidal injection of triamcinolone in the right eye to treat CME after discussing potential benefits and complications with the patient. The 30-gauge needle limits delivery to 1000 microns at the pars plana, with detailed assembly methods provided in previous publications and a video (Fig. 4).^[11–15]^ The ocular area was sanitized with 10% povidone-iodine, and the conjunctiva was treated with 4% povidone-iodine. Under topical anesthesia, an injection site was marked 4 mm from the limbus in the superior-temporal quadrant. A custom needle was used to deliver 0.1 mL of triamcinolone acetonide (4 mg) into the suprachoroidal space, ensuring proper technique to avoid resistance and reflux. Visual acuity and vein pulsation were assessed to rule out retinal artery occlusion. Any increase in intraocular pressure was managed via paracentesis. Postinjection, pupil dilation facilitated examination for triamcinolone penetration or signs of complications such as detachment or hemorrhage. Due to advanced atrophy, treatment options for the left eye were not available.

**Figure 5.**
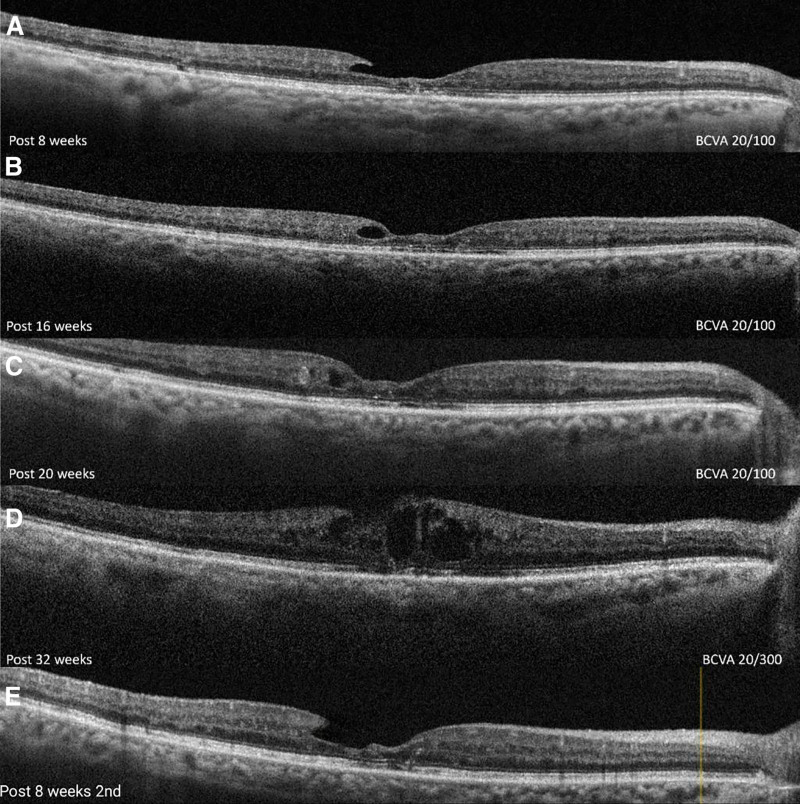
(A) The OCT image of the right eye after 8 wk of suprachoroidal triamcinolone injection shows resolved cystic changes. (B) The OCT image of the right eye after 16 wk of suprachoroidal triamcinolone injection shows the formation of a new cyst. (C) The image of the right eye after 20 wk of suprachoroidal triamcinolone injection shows stability of the cyst with no increase in retinal thickness. (D) The OCT image of the right eye after 32 wk of suprachoroidal triamcinolone injection shows a severe increase in retinal thickness with the formation of new cysts. (E) The OCT image of the right eye after 8 wk of the second suprachoroidal triamcinolone injection shows complete resolution of all cysts with normalization of retinal thickness. OCT = optical coherence tomography.

After 8 weeks post, her best-corrected visual acuity (BCVA) improved to 20/100, and OCT showed resolved cysts. Follow-up OCT scans at 16 weeks and 32 weeks indicated the emergence of new cysts, with stable BCVA maintained at 20/100. However, by 32 weeks postinjection, her BCVA declined to 20/300. A second suprachoroidal injection of triamcinolone was administered, and after an additional 8 weeks of follow-up, her BCVA returned to 20/100 with resolved cysts (Fig. 5). Subsequent follow-ups revealed no signs of regression. At the 6-month follow-up, there was no increase in intraocular pressure; however, a posterior subcapsular cataract developed as a result of the triamcinolone injections and was subsequently treated with phacoemulsification.

## 4. Discussion and conclusions

LMH was first described by Gass in 1975 as a complication of CME.^[16]^ It is proposed to differentiate LMH into 2 subtypes: tractional LMH (ERM foveoschisis) and degenerative LMH (true LMH).^[4]^ Some studies suggest that tractional LMH should be excluded from the LMH category, as they are clinically, morphologically, and pathogenically distinct.^[17]^ Conversely, other research indicates that both ERMF and LMH may arise from a tractional event, representing essentially the same condition at different stages of development.^[4]^ LMH associated with CME is characterized as degenerative. This characterization likely results from the tractional stretching and thickening of the foveal center, which is caused by a foveal pseudocyst. This phenomenon occurs following the detachment of the foveola from the retinal pigment epithelium, leading to a disruption of the foveal structure due to CME.^[18]^

Currently, there are no pharmacological treatments available for LMH; therefore, surgical intervention remains the primary approach. This typically involves a procedure known as pars plana vitrectomy (PPV), which may include the removal of preretinal tissue (ERM/ERP) and/or the internal limiting membrane.^[4]^ These surgical procedures are associated with a high rate of complications. In a retrospective study by Casparis et al, 44 patients with LMH who underwent PPV and ERP demonstrated an improvement in BCVA from 0.4 to 0.13. However, 53% of these patients developed cataracts.^[19]^ Another retrospective study by Figueroa et al, involving 12 patients with LMH who underwent PPV, found that 25% of them developed a full-thickness macular hole (FTMH).^[20]^

Despite these findings, previous research did not provide detailed characteristics of LMH in their study subjects, making it challenging to predict surgical outcomes for degenerative LMH.^[17]^ In a study conducted by Coassin et al, which included 106 patients – 65% with tractional LMH and 35% with degenerative LMH undergoing PPV with ERM – it was found that BCVA did not improve in the degenerative LMH group.^[21]^ Degenerative LMH is associated with poorer functional and morphological outcomes and carries a risk of progression to FTMH. As such, surgical intervention may not be the most appropriate treatment approach for this condition. Therefore, there is an urgent need for further research to better understand the characteristics of degenerative LMH and to identify the most effective therapeutic options for its management.^[17]^

The positioning of the suprachoroidal space results in a slower elimination rate and increased absorption of medication into specific target tissues. Research has extensively evaluated the use of suprachoroidal triamcinolone injections for treating DME associated with RVO and diabetes, demonstrating both effectiveness and safety. However, potential complications include elevated intraocular pressure (IOP) and cataract formation, with a reported cataract incidence of 9.1%.^[10]^ In our case, the patient developed a posterior subcapsular cataract after the second injection; however, there was no elevation in IOP.

In this study, suprachoroidal injections of triamcinolone were employed to manage CME and enhance the patient’s BCVA. After treatment, OCT revealed the resolution of the LMH following the initial injection, and the patient subjectively reported improved vision. However, at the 24-week follow-up, LMH progression necessitated a subsequent injection, which effectively resolved the condition and sustained visual improvement. The mechanisms underlying the effectiveness of this treatment may stem from 1 or more of the following factors: first, the resolution of CME, which leads to decreased intraretinal traction and restoration of foveal contour; second, steroid-mediated suppression of inflammatory cytokines that contribute to retinal remodeling; or third, enhanced retinal adhesion resulting from fluid resorption. The author utilized a custom-designed needle developed specifically for these injections, offering a cost-effective and less invasive alternative to surgical intervention. Nevertheless, it is crucial to acknowledge potential limitations, as improper administration of suprachoroidal injections may lead to choroidal detachment or hemorrhage. Furthermore, the fixed infiltration depth of the needle raises concerns regarding the risk of inadvertent triamcinolone injection into the vitreous cavity, particularly in patients with thin sclera.

The administration of suprachoroidal triamcinolone acetonide via a custom-made needle may serve as a viable treatment option for lamellar holes associated with CME resulting from RVO. This approach has demonstrated promising outcomes with fewer complications and lower costs compared to traditional surgical interventions. However, it is important to note that this study is based solely on a single case report without a control group. Therefore, further research is necessary to validate our preliminary findings regarding the efficacy and safety of this treatment method. Additionally, the findings are limited by several factors that affect their generalizability, including a lack of long-term follow-up, absence of quantitative OCT metrics such as central retinal thickness, and the inability to assess reproducibility or compare against standard treatments like vitrectomy or intravitreal steroids.

## Author contributions

**Data curation:** Sedra Abu Ghedda.

**Formal analysis:** Sedra Abu Ghedda, Ameen Marashi.

**Investigation:** Marwa Baba.

**Resources:** Ameen Marashi.

**Software:** Marwa Baba, Ameen Marashi.

**Validation:** Reem Farwati, Marwa Baba.

**Writing – original draft:** Sedra Abu Ghedda.

**Writing – review & editing:** Sedra Abu Ghedda, Reem Farwati.
